# Insufficient nutrient intake in individuals with disabling hearing loss and the restoration of nutritional sufficiency in hearing aid users

**DOI:** 10.1038/s41598-024-57927-w

**Published:** 2024-03-29

**Authors:** Sang-Yoon Han, Sang-Yeon Lee, Myung-Whan Suh, Jun Ho Lee, Moo Kyun Park

**Affiliations:** 1 Department of Otolaryngology-Head and Neck Surgery, College of Medicine, Hanyang University, Seoul, Republic of Korea; 2 Department of Otorhinolaryngology-Head and Neck Surgery, Seoul National University Hospital, 101 Daehak-ro, Jongno-gu, Seoul, Republic of Korea; 3 Sensory Organ Research Institute, Medical Research Center, Seoul National University, Seoul, Republic of Korea

**Keywords:** Hearing loss, Nutrient, Malnutrition, Health care, Medical research, Risk factors, Diseases, Nutrition disorders, Malnutrition, Disability

## Abstract

Hearing loss affects some nutrient intake. Disabling hearing loss may exacerbate these issues. We aimed to evaluate nutrient intake and assess deficiencies based on functional hearing status. The study included 6907 participants with information on demographic factors, nutrient intake, weight, height, disease status, and hearing level in the eighth Korea National Health and Nutrition Examination Survey, conducted from 2019 to 2021. We categorized the participants into 3 groups based on their functional hearing status: bilateral hearing, unilateral hearing, and disabling hearing loss. The disabling hearing loss group showed lower intake of most major nutrients (*P* < 0.05), dietary fiber (*P* < 0.001), and most minerals and vitamins (*P* < 0.05), with some insufficiencies. The unilateral hearing group showed lower intake only for potassium (*P* = 0.036) compared to the bilateral hearing group and significantly higher intake of hydration (*P* = 0.039), dietary fiber (*P* = 0.039), and calcium (*P* = 0.009) than the disabling hearing loss group. Nutrient insufficiency in the disabling hearing loss group was more prominent in women, and was partially resolved by using hearing aids. Clinicians and nutritionists should consider undernourishment in these patients, and appropriate interventions for nutrition and hearing aids should be recommended.

## Introduction

Hearing loss in middle-aged and older adults is a significant problem for their physical and mental health^1,2^. Martinez-Amezcua and Powell et al. have demonstrated that hearing loss is significantly associated with poor physical health^1,2^. Moreover, Lin and Ferrucci have reported a relationship between hearing loss and the rate of falls^3^. Furthermore, some studies have found significantly higher mortality in patients with hearing loss^4,5^. Hearing loss also affects mental health, leading to issues such as anxiety and depression^6,7^.

Additionally, hearing status may influence food and nutrient intake^8,9^. Some studies have linked hearing loss to a reduced intake of essential nutrients, including vitamins, zinc, magnesium, and omega-3 fatty acids^9–12^. Jung et al.^9^ conducted a systematic analysis of the association between hearing loss and nutrient intake. Their research indicated that previous studies had identified associations between hearing loss and the intake of certain nutrients^9^. These associations may be due to inadequate antioxidant intake, which can induce hearing-related issues, or to the impaired physical activity and mental health of individuals with hearing problems^2,7,13–20^. However, these earlier studies simply reported lower consumption of specific nutrients, including vitamins, n-3 fatty acids, magnesium, and zinc, in individuals with hearing loss^8,9,21,22^. Additionally, these studies often characterized the nutrients linked to hearing loss as risk factors, or they restricted their analysis to cases of mild hearing loss^8,9,21,22^. Moreover, these studies used different definitions of hearing loss. One defined it as a bilateral mean hearing level exceeding 40 dB^10^, while another considered a unilateral hearing level above 25 dB as indicative of hearing loss.^12^ Moreover, most research in this area has been based on the average hearing thresholds of both ears or has only taken into account the hearing levels of the better ear^8,9,21,22^.

Hearing loss is categorized into various degrees according to its severity. A diagnosis is made when a person's hearing threshold exceeds 25 dB^23,24^. Individuals with mild hearing loss can generally hear well enough to engage in conversation and participate in social activities^23^. Therefore, hearing aids are often recommended for individuals whose hearing loss is greater than 40 dB^23,25^. However, hearing aids are not typically used for unilateral hearing loss, as they can be uncomfortable or the benefits may not be perceived as significant^26–29^. Although individuals with unilateral functional hearing may experience some difficulties compared to those with normal hearing, these issues are not as profound as those encountered by individuals with disabling hearing loss^23^. In contrast, individuals with bilateral moderate hearing loss often face significant challenges in communication and usually rely on hearing aids^23^.

Consequently, disabling hearing loss is associated with stronger physical and psychological effects^25^. Since hearing loss is associated with the intake of some nutrients, disabling hearing loss may exacerbate these issues to a greater extent than mild or unilateral hearing loss. The objective of this study was to evaluate nutritional intake in relation to the hearing status of each ear, categorized as either functional or non-functional. Our aim was to assess the nutritional status, taking into account gender differences, among individuals with functional hearing in one or both ears compared to those without, using the 2020 Dietary Reference Intakes for Koreans (KDRI) as a reference^30^.

## Materials and methods

### Subjects and data extraction

**Figure 1:**
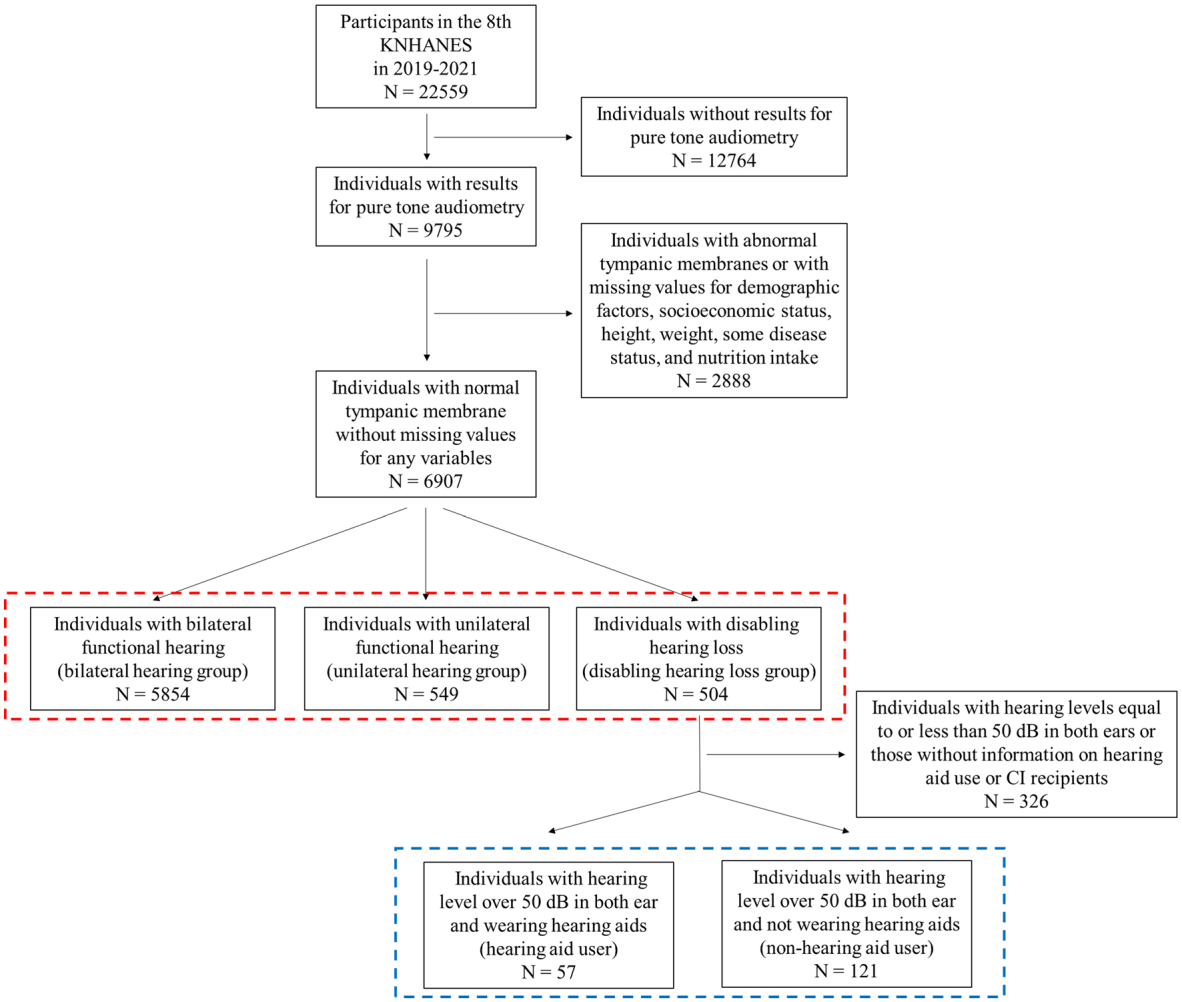
A flow chart of subject selection for the analysis of the association between hearing loss and nutritional intake (depicted in red) and between hearing aid usage and nutritional intake (depicted in blue). KNHANES, Korea National Health and Nutrition Examination Survey; N, number of participants; CI, cochlear implant.

We extracted data on participants in the eighth Korea National Health and Nutrition Examination Survey (KNHANES), which was conducted from 2019 to 2021. The survey comprised 22,559 participants. These participants were selected using two-stage stratification and random sampling methods to prevent selection bias. Of these, 9795 individuals who had undergone pure tone audiometry were selected. Audiometry was performed exclusively on individuals over 40 years old in the eighth KNHANES, considering the prevalence of hearing loss, which sharply increases with age, particularly in individuals aged over 40^31^. Therefore, only middle-aged and older adults were included in our study. Subsequently, we narrowed down the cohort to those with normal tympanic membranes and complete data on gender, age, economic status, education level, height, weight, nutritional intake, and medical history, specifically regarding hypertension, dyslipidemia, stroke, coronary artery disease, diabetes mellitus, major depressive disorder, otitis media, chronic kidney disease, gout, and liver cirrhosis. The information on nutritional intake was collected by the investigators, who visited households and evaluated food intake based on participants' recall for the 24 h preceding the survey. Ultimately, 6907 participants met the criteria and were included in this study (Fig. 1). All methods used in the eighth KNHANES survey and our study were carried out in accordance with the relevant guidelines and regulations, and our study was conducted based on the STROBE statement. The eighth KNHANES survey was conducted after obtaining informed consent from all participants and receiving approval from the Institutional Review Board (IRB No. 2018-01-03-C-A, 2018-01-03-2C-A, 2018-01-03-5C-A).

### Classification of the participants according to their hearing status

Disabling hearing loss is characterized by a hearing loss greater than 40 dB in the better ear. The term “functional hearing” refers to a hearing level below 40 dB, differentiating it from disabling hearing loss. Participants were categorized into three groups according to their functional hearing status: the bilateral hearing group, which had bilateral hearing levels below 40 dB; the unilateral hearing group, which had functional hearing in one ear and more than moderate hearing loss (> 40dB) in the other; and the disabling hearing loss group, which had a bilateral hearing loss exceeding 40 dB.

### Nutritional status evaluation

We utilized the KDRI as the benchmark for determining insufficient intake of each nutrient. The KDRI provides guidelines for the estimated average requirement (EAR), recommended dietary allowance (RDA), and adequate intake (AI). We assessed the insufficiency of nutrient intake against these references.

### Subgroup analysis of differences in nutrient intake based on the use of hearing aids

Since hearing loss patients typically use hearing aids when their hearing level is around 50 dB, and patients with hearing thresholds below 50 dB usually exhibit a tendency to refuse the use of hearing aids,^32,33^ we selected patients with hearing loss greater than 50 dB. Subsequently, we included patients with information about hearing aid use, excluding the three patients who had undergone cochlear implantation (Fig. 1).

### Statistical analysis

Analysis of variance and the chi-square test were utilized to compare demographic and socioeconomic factors, as well as the prevalence of medical history, across the study groups. Following this, multivariate analysis of covariance (MANCOVA) was conducted to determine the adjusted average and standard error, taking into account variables such as gender, age, weight, height, and medical history. The MANCOVA calculations were also carried out separately for male and female participants. The adjusted mean and standard error for each group, along with the KDRI values, were subsequently normalized by dividing them by the adjusted mean value for the total cohort. This normalization process was designed to evaluate nutritional insufficiency by creating a ratio that reflects the average intake relative to that of the entire study population. All statistical analyses were performed using IBM SPSS Statistics 25.0 (IBM Corp., Armonk, N.Y., USA).

## Results

### Demographics, socioeconomic status, physical examination results, and medical history of each group

There were 5854, 549, and 504 participants in the bilateral hearing group, unilateral hearing group, and disabling group, respectively (Fig. 1).

Age, gender, household income, education level, weight, and height differed significantly among the groups. Furthermore, the prevalence rates of hypertension, dyslipidemia, stroke, coronary artery disease, diabetes mellitus, and previous history of otitis media also showed significant differences across the groups (Table 1).

**Table 1:** Demographic factors, socioeconomic factors, height, weight, and medical history of each group.

	Bilateral hearing group (N = 5854)	Unilateral hearing group (N = 549)	Disabling hearing loss group (N = 504)	*P*-value
Age	57.5 ± 10.9	68.5 ± 9.5	73.1 ± 7.7	** < 0.001**
Gender (male:female)	2327:3527	295:254	283:221	** < 0.001**
Household income (quintile)	3.3 ± 1.4	2.5 ± 1.3	2.1 ± 1.2	** < 0.001**
Education level (lower than high school)	29.7% (1737/5854)	59.0% (324/549)	69.8% (352/504)	** < 0.001**
Height	162.2 ± 8.8	160.7 ± 9.0	160.0 ± 9.3	** < 0.001**
Weight	64.0 ± 11.9	63.1 ± 11.2	61.4 ± 10.8	** < 0.001**
Hypertension	29.6% (1732/5854)	48.1% (264/549)	57.9% (292/504)	** < 0.001**
Dyslipidemia	28.6% (1676/5854)	39.2% (215/549)	33.9% (171/504)	** < 0.001**
Stroke	2.5% (144/5854)	5.3% (29/549)	8.5% (43/504)	** < 0.001**
Coronary artery disease	3.7% (216/5854)	6.9% (38/549)	7.3% (37/504)	** < 0.001**
Diabetes mellitus	12.1% (706/5854)	23.0% (126/549)	27.2% (137/504)	** < 0.001**
Major depressive disorder	5.5% (322/5854)	5.5% (30/549)	6.7% (34/504)	0.501
Otitis media (past medical history)	3.2% (186/5854)	6.6% (36/549)	5.4% (27/504)	** < 0.001**
Chronic kidney disease	1.6% (95/5854)	2.2% (12/549)	3.0% (15/504)	0.064
Gout	2.5% (146/5854)	1.8% (10/549)	2.8% (14/504)	0.204
Liver cirrhosis	0.4% (23/5854)	0.7% (4/549)	0.4% (2/504)	0.507

Significant values are in [bold].

### Nutritional intake differences among the groups

**Figure 2:**
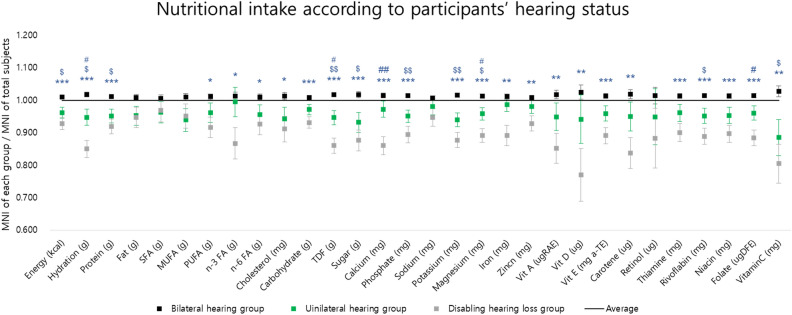
The age- and gender-adjusted nutrient intake of each group. MNI, mean nutrient intake; SFA; saturated fatty acid, MUFA; monounsaturated fatty acid, PUFA; polyunsaturated fatty acid, n-3-FA; n-3 polyunsaturated fatty acid, n-6 FA, N-6 polyunsaturated fatty acid; TDF, total dietary fiber; Vit, vitamin; *statistically meaningful difference between the bilateral hearing group and the disabling hearing loss group; *, *P* < 0.05; **, *P* < 0.01; ***, *P* < 0.001; $, statistically meaningful difference between the bilateral hearing group and the unilateral hearing group; $, *P* < 0.05; $$, *P* < 0.01; $$$, *P* < 0.001; #, statistically meaningful difference between the unilateral hearing group and the disabling hearing loss group; #, *P* < 0.05; ##, *P* < 0.01; ###, *P* < 0.001.

In an age- and gender-adjusted analysis, significant differences were observed among the groups in their intake of energy, hydration, protein, polyunsaturated fatty acids, omega-3 fatty acids, omega-6 fatty acids, cholesterol, carbohydrates, total dietary fiber (TDF), sugar, calcium, phosphate, potassium, magnesium, iron, zinc, vitamin A, vitamin D, vitamin E, carotene, thiamine, riboflavin, niacin, folate, and vitamin C. The results of the post-hoc tests are presented in Fig. 2.

**Figure 3:**
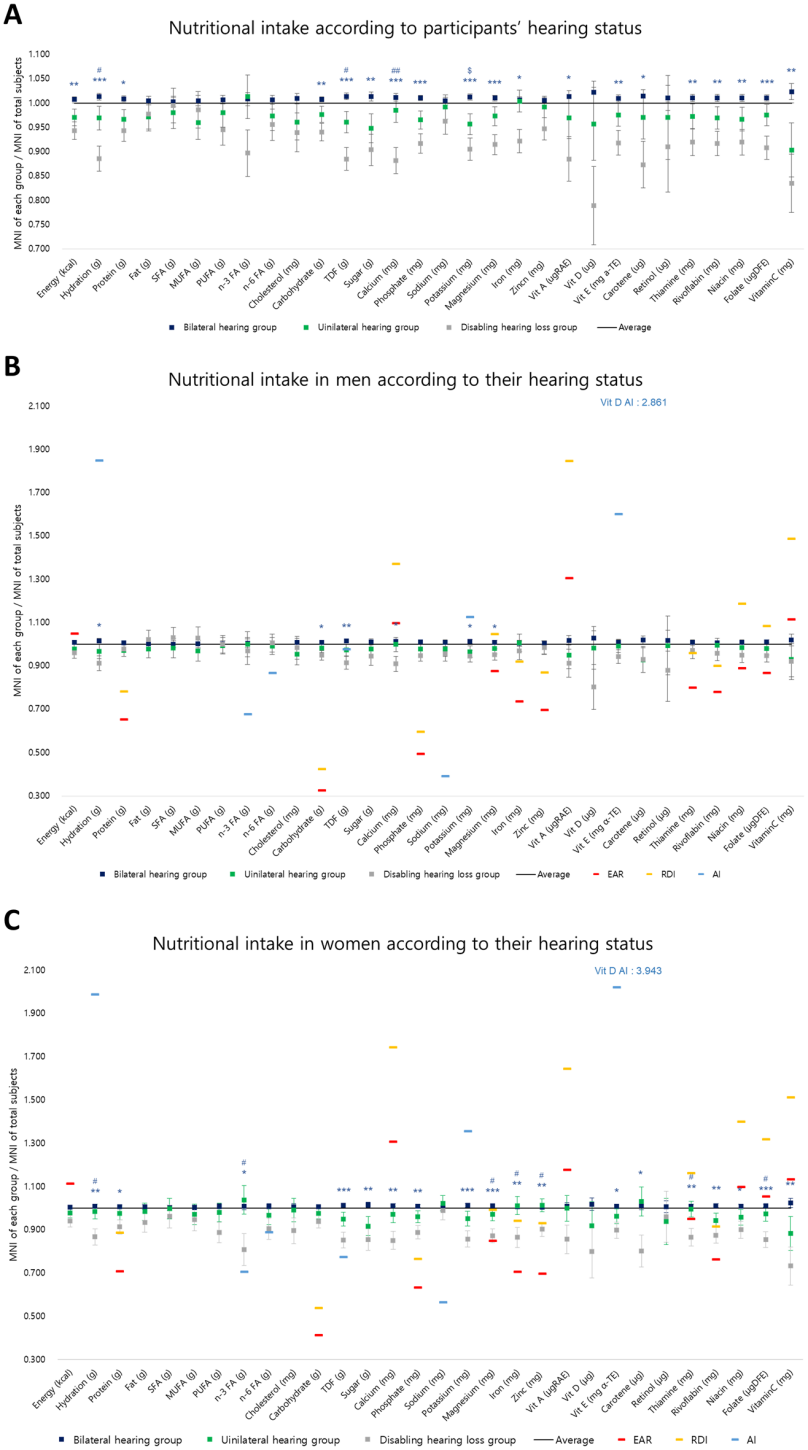
The nutrient intake of each group for total (**A**), male (**B**), and female (**C**) participants and the dietary reference intake values for male (**B**) and female (**C**) populations after controlling other factors. MNI, mean nutrient intake; EAR, estimated average requirement; RDI, recommended daily intake; AI, adequate intake; SFA, saturated fatty acid; MUFA, monounsaturated fatty acid; PUFA, polyunsaturated fatty acid; n-3-FA, n-3 polyunsaturated fatty acid; n-6 FA, N-6 polyunsaturated fatty acid; TDF, total dietary fiber; Vit, vitamin; * statistically meaningful difference between the bilateral hearing group and the disabling hearing loss group; *, *P* < 0.05; **, *P* < 0.01; ***, *P* < 0.001; $, statistically meaningful difference between the bilateral hearing group and the unilateral hearing group; $, *P* < 0.05; $$, *P* < 0.01; $$$, *P* < 0.001; #, statistically meaningful difference between the unilateral hearing group and the disabling hearing group; #, *P* < 0.05; ##, *P* < 0.01; ###, *P* < 0.001.

After adjusting for age, gender, household income, educational status, height, weight, hypertension, dyslipidemia, stroke, coronary artery disease, diabetes mellitus, and previous history of otitis media, significant differences were observed in the intake of energy, hydration, protein, carbohydrates, TDF, sugar, calcium, phosphate, potassium, magnesium, iron, vitamin A, vitamin D, vitamin E, beta-carotene, thiamine, riboflavin, niacin, folate, and vitamin C among the groups. Post-hoc analysis revealed that all nutrient intake levels that differed significantly between groups were lower in the disabling hearing loss group compared to the bilateral hearing loss group. The unilateral hearing loss group had lower potassium intake than the bilateral hearing loss group. Additionally, the unilateral hearing loss group had significantly higher intake of hydration, TDF, and calcium compared to the disabling hearing loss group, as shown in Fig. 3A.

### Differences in nutrient intake among the groups of male participants

A subgroup analysis was conducted for the male population. After adjusting for other factors, we observed differences in nutrient intake between the groups, which included hydration, carbohydrates, TDF, calcium, and potassium. The intake levels of all these nutrients were greater in the bilateral hearing group compared to the disabling hearing loss group except for potassium. No differences in nutrient intake were noted between the bilateral and unilateral hearing groups (Fig. 3B).

### Differences in nutrient intake among the groups of female participants

In female participants, the consumption of energy, hydration, protein, omega-3 fatty acids, TDF, sugar, calcium, phosphate, potassium, magnesium, iron, zinc, vitamin E, carotene, thiamine, riboflavin, niacin, folate, and vitamin C differed significantly among the groups after adjusting for other factors. In the post-hoc analysis, the bilateral hearing group showed higher nutrient intake compared to the disabling hearing loss group, with the exception of energy. The intake of hydration, omega-3 fatty acids, magnesium, iron, zinc, thiamine, and folate was also notably lower in the disabling hearing loss group compared to the unilateral hearing group. No significant differences were observed in nutrient intake between the bilateral and unilateral hearing groups (Fig. 3C).

### Nutrient status

#### Male participants

The calcium, vitamin A, and vitamin C intake of all groups of male participants did not meet the EAR (Fig. 3B). Additionally, all groups showed insufficient intake of magnesium, niacin, and folate based on the RDI (Fig. 3B). Moreover, the intake of hydration, potassium, vitamin D, and vitamin E was lower than the AI in all groups. The TDF intake was lower than the AI in the unilateral hearing group and the disabling hearing loss group (Fig. 3B).

#### Female participants

All groups of female participants showed insufficient intake of energy, calcium, vitamin A, niacin, folate, and vitamin C based on the EAR. Additionally, the disabling hearing loss group exhibited insufficient thiamine intake based on this criterion (Fig. 3C). Potassium and thiamine intake was also lower than the RDI values in all groups. Furthermore, the disabling hearing loss group showed deficient magnesium, iron, zinc, and riboflavin intake based on the RDI (Fig. 3C). Additionally, hydration, vitamin D, and vitamin E intake did not meet the AI in all groups (Fig. 3C).

### Differences in nutrient intake based on the use of hearing aids

There were 57 hearing aid users and 121 non-hearing aid users (Fig. 1). Among the analyzed factors—namely, age, gender, household income, education level, weight, height, medical history, and bilateral mean hearing level calculated by averaging the pure tone audiometry at 0.5 kHz, 1 kHz, 2 kHz, and 4 kHz— the educational level (*P* = 0.012) and mean hearing level (*P* = 0.001) exhibited statistically significant differences between the hearing aid users and non-users.

**Figure 4:**
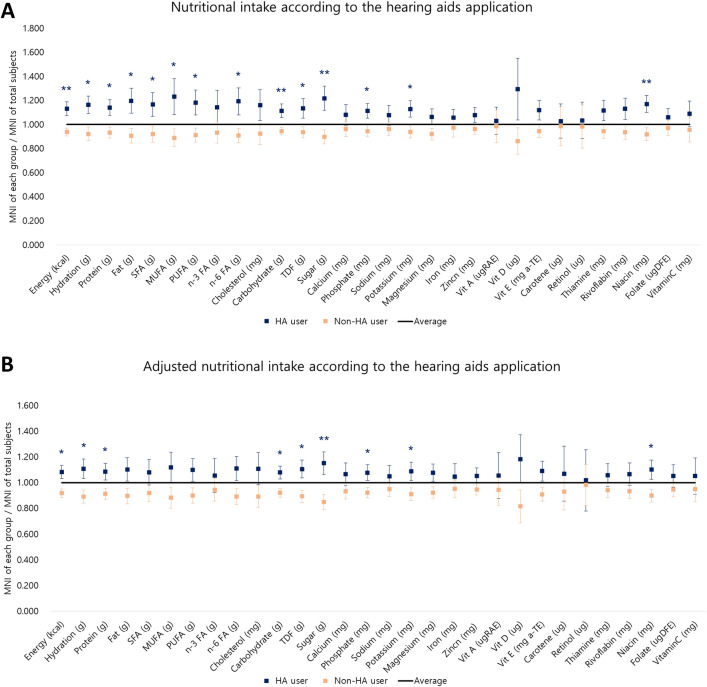
The nutrient intake of hearing aid users and non-users in univariable (**A**) and multivariable analysis (**B**). MNI, mean nutrient intake; HA, hearing aids; SFA, saturated fatty acid; MUFA, monounsaturated fatty acid; PUFA, polyunsaturated fatty acid; n-3-FA, n-3 polyunsaturated fatty acid; n-6 FA, N-6 polyunsaturated fatty acid; TDF, total dietary fiber; Vit, vitamin; * statistically meaningful difference between the hearing aids user and the non-hearing aid user; *, *P* < 0.05; **, *P* < 0.01; ***, *P* < 0.001.

The hearing aid users also showed significantly higher intake of energy (*P* = 0.002), hydration (*P* = 0.013), protein (*P* = 0.01), fat (*P* = 0.01), saturated fatty acids (*P* = 0.047), monounsaturated fatty acids (*P* = 0.023), polyunsaturated fatty acids (*P* = 0.015), omega-6 fatty acids (*P* = 0.027), carbohydrates (*P* = 0.005), TDF (*P* = 0.024), sugar (*P* = 0.004), phosphate (*P* = 0.03), potassium (*P* = 0.031), and niacin (*P* = 0.007) (Fig. 4A).

After adjusting for the education level and mean hearing level, hearing aid users exhibited higher intake of energy (*P* = 0.01), hydration (*P* = 0.021), protein (*P* = 0.032), carbohydrates (*P* = 0.01), TDF (*P* = 0.014), sugar (*P* = 0.006), phosphate (*P* = 0.041), potassium (*P* = 0.043), and niacin (*P* = 0.03) (Fig. 4B).

## Discussion

Hearing loss is associated with a range of functions and health conditions, including physical and mental health, nutritional intake, and economic status^4–6,8,9,21,22,34–37^. We categorized individuals by their functional hearing status to assess differences in nutritional intake across these groups. Our findings showed that varied aspects of nutritional intake were correlated with the degree of disabling hearing loss. Specifically, the quantity of nutrient consumption decreased progressively with worsening functional hearing status in middle-aged and older adults, even after adjusting for other variables. Nutrient intake levels, including hydration, protein, carbohydrates, TDF, sugar, calcium, phosphate, potassium, magnesium, iron, vitamin A, vitamin D, vitamin E, the vitamin B complex, and vitamin C, were notably lower in the group with disabling hearing loss compared to those with bilateral hearing, as determined by multivariable analysis. Furthermore, nutritional deficiencies were more pronounced among women with disabling hearing loss. The group with unilateral hearing loss had higher consumption of some nutrients compared to those with disabling hearing loss and exhibited certain differences from the bilateral hearing group in age- and gender-adjusted analysis. However, these differences in nutrient intake were not statistically significant in the unilateral hearing group relative to the bilateral hearing group after adjusting for additional factors.

Moreover, the intake of hydration, calcium, potassium, magnesium, vitamin A, vitamin D, vitamin E, niacin, folate, and vitamin C did not meet the KDRI in all male groups. Additionally, the TDF intake was below the KDRI recommendations for both the unilateral hearing loss group and the disabling hearing loss group. Female participants also demonstrated inadequate intakes of energy, hydration, calcium, vitamin A, vitamin D, vitamin E, thiamine, niacin, folate, and vitamin C according to the KDRI. Moreover, women with disabling hearing loss had insufficient intake of magnesium, iron, zinc, thiamine, and riboflavin, according to the KDRI guidelines. The prevalence of nutrient insufficiency was significantly higher in the groups with disabling hearing loss for both men and women. Consequently, individuals in the disabling hearing loss group were at an increased risk of nutritional deficiencies, including hydration, energy, and various essential minerals and vitamins.

There were no significant differences in the intake of most nutrients between the bilateral hearing group and the unilateral hearing group upon multivariable analysis. Potassium, which did show variations between the two groups, was found to be consumed in lower level than AI by both. The other nutrient deficiencies observed in the unilateral hearing group, compared to the bilateral functional group, were for TDF and calcium, according to the KDRI. Nonetheless, the differences in intake levels between the bilateral and unilateral hearing groups were not statistically significant. Consequently, the unilateral hearing group demonstrated a slightly insufficient nutrient status, akin to that of the bilateral hearing group. The reason for the less meaningful association between unilateral hearing and nutritional intake is the slight degree of functional loss in the unilateral hearing group. Although individuals with unilateral functional hearing may experience some functional loss compared to those with normal hearing, these issues are not as profound as those encountered by individuals with disabling hearing loss^23,25^. In contrast, the disabling hearing loss group exhibited significant differences in the intake of many nutrients, with some levels falling below the recommended thresholds.

Energy deficiency can induce low lean mass^38^. Inadequate hydration has been linked to metabolic impairment, degenerative diseases, decreased kidney function, impaired skin barrier function, and cognitive impairment^39^. Since calcium is a key factor for osteogenesis, calcium metabolism, and preventing osteoporosis, sufficient calcium intake is necessary for middle-aged and older adults^40^. Additionally, calcium deficiency has been associated with age-related diseases such as atherosclerosis, neurodegenerative diseases, degenerative arthritis, and malignancy^40^. Magnesium is the second most common intracellular cation, and it is essential for energy production and nucleic acid synthesis^41^. It has also been linked to various symptoms and diseases, including asthma, osteoporosis, migraine, metabolic syndrome, and muscle cramps^41^. Iron plays a pivotal role in hematopoiesis and is important in preventing anemia^42^. Zinc is crucial for growth and reproduction,30 and is necessary for numerous physiological processes. Deficiency in zinc can lead to dermatitis, impaired growth, and an increased risk of infection^41^. Vitamin A is important for eye function and the immune system^43–46^. Deficiency in vitamin A can manifest as eye signs such as night blindness, conjunctival xerosis, corneal xerosis, and eye ulcers^44,46^. Vitamin D is very important for preventing rickets, osteoporosis, and neurodegenerative diseases^47–49^. Vitamin E acts as an antioxidant, scavenging reactive oxygen species, and plays a role in immunomodulation^50^. Its deficiency has been linked to infections and growth problems^51^. The vitamin B complex is associated with megaloblastic anemia, physical function, and central nervous system functions, including cognitive decline, depression, confusion, epileptiform convulsions, and Wernicke-Korsakoff syndrome in cases of severe thiamine deficiency^52^. Additionally, vitamin C serves as an antioxidant and as a cofactor for certain enzymes, such as hydroxylase, which is necessary for collagen synthesis^53^. Recent studies have suggested that vitamin C may have a role in the prevention or treatment of some infections^54^. Furthermore, a severe deficiency of vitamin C can result in scurvy^53^.

These essential and significant nutrients were found to be more deficient in participants with disabling hearing loss than in either of the other two groups. This deficiency could lead to a higher prevalence of physical and mental problems. Moreover, research by Le Prell et al., has shown that intake of magnesium and vitamins A, E, and C can prevent noise-induced hearing loss by neutralizing free radicals, as demonstrated in animal studies. Additionally, several prospective and randomized controlled studies have indicated that consumption of omega-3 fatty acids, magnesium, and vitamins A, E, B, and C may help prevent hearing loss^13–18^. Consequently, nutrient insufficiency in patients with hearing loss can exacerbate the condition and negatively impact overall health. Therefore, nutritional interventions, including supplements or dietary consultations, should be considered essential for those with disabling hearing loss to prevent further hearing loss as well as other health complications. Such measures could play a crucial role in preserving both the physical and mental well-being of these patients.

Nutrient insufficiency can also occur in association with other diseases. A previous study examining the nutritional status of individuals with depression found that these patients often have deficiencies in protein and energy, as well as lower intakes of folate and vitamin B12, when compared to recommended dietary allowances^55^. Similarly, research by Weikel et al. indicated that patients with cataracts commonly exhibit deficiencies in vitamins B and C, lutein/zeaxanthin, omega-3 fatty acids, multivitamins, and carbohydrates, relative to recommended nutrient levels^56^. Similarly, our research found that patients with disabling hearing loss were at risk of inadequate nutrient intake. Investigating the impact of various disorders on nutritional status is crucial to prevent additional complications arising from the primary diseases.

Differences in nutrient intake among the groups were observed in the consumption of some major nutrients, some fatty acids, various vitamins, and minerals. Conversely, no differences were noted in the intake of fat, saturated fatty acids (SFA), cholesterol, and sodium. Generally, fat, SFA, cholesterol, and sodium are considered unhealthy nutrients. These findings align with a previous study by Choi et al.^22^, which reported that fruit consumption in the bilateral hearing loss group was lower than that in the normal hearing group. In addition, our results are consistent with previous studies that have reported associations between omega-3 fatty acids, minerals, or vitamins and hearing loss^8,9,21,22^. Therefore, individuals with disabling hearing loss seem to consume fewer healthy foods and a greater proportion of unhealthy foods compared to those with bilateral functional hearing. This pattern of nutrient intake should be considered when developing dietary interventions.

In this study, insufficient nutrient intake among the female population appeared to be more closely linked to disabling hearing loss. Kushwaha et al.^57^ reported that women were more prone to malnutrition. Schilp et al.^58^ also demonstrated a significant association between the female gender and malnutrition. Additionally, women tend to be more susceptible to functional loss associated with hearing loss^7,20^. Previous studies have reported that female hearing loss patients experienced higher levels of depression and loneliness than male hearing loss patients^7,20^. Our results are consistent with these tendencies. Therefore, nutritional interventions should be particularly targeted toward female patients with disabling hearing loss.

Compared to previous studies on the association between hearing loss and nutrient intake^8,9^ , we classified individuals as having disabling hearing loss or functional hearing. Since disabling hearing loss critically affects other functions^23,25,59^, the differences in nutrient intake among the groups might be more prominent in our study than in previously conducted studies.

Additionally, the use of hearing aids proved helpful in compensating for nutritional insufficiency in individuals with bilateral disabling hearing loss, exceeding 50 dB. Given that hearing aids can mitigate some adverse consequences of hearing loss, including enhancing social network status and alleviating depressive symptoms^6,35,36^, they may influence nutritional status. Additional research is needed to elucidate the effect of hearing aids on nutrient intake.

The participants in this survey were selected using two-stage stratification and random-sampling methods to prevent selection bias. However, despite the short-term recall periods before the survey, as the data on nutritional intake were collected through participant recall, the potential for recall errors existed. Prospective nutritional data collection would be helpful in preventing these kinds of errors in future studies.

Since this study employed a cross-sectional design, it was not possible to evaluate the causality between disabling hearing loss and nutrient intake. Emmett et al.^37^ found that malnutrition during childhood can impact the hearing levels in young adults. Because nutrient deficiency can induce hearing loss, a prospective cohort study could be instrumental in demonstrating the causality of this association.

Another limitation of our study is the lack of information on other audiological exams, such as bone conduction hearing levels or speech audiometry. Since the KNHANES did not include other audiological examinations, we could not evaluate the association of other audiological results with nutritional intake. A further study, including speech audiometry, may be helpful for assessing the relationship between daily speech recognition ability and nutrient intake.

## Conclusion

Individuals with disabling hearing loss are susceptible toseveral significant nutrient deficiencies, and this tendency was more pronounced in women. Given the possibility of undernourishment in patients with disabling hearing loss, hearing aid use and appropriate nutritional interventions, including nutritional consultation or supplementation, could enhance the physical and mental health of this patient population.
